# 

**Published:** 1903-11-07

**Authors:** 


### CONTENTS.

**Feeding and Disease** - 93

**The Elimination of the Inebriate** - 94

**Annotations** - 95

**Notes and News** - 96

**Hospital Clinics and Medical Progress—**

The Care of Rheumatic Children - 97

Difficulty in Swallowing - 98

Dislocation of the Outer End of the Clavicle - 98

The Use of Copper in Syphilis - 99

Perforation of the Colon by Small Foreign Bodies - 99

Progress in Medicine - 99

Progress in Psychiatry - 101

Respiratory Diseases - 102

**New Appliances and Things Medical** - 103

**Modern Sociology** - 104

**Hospital Administration—**

Modern Hospital and Institutional Fittings.—II. - 105

Rise and Growth of Vaccination Law.—III. - 106

The Kelling Sanatorium, Holt, Norfolk - 107

The King Edward VII. Sanatorium - 107

**The Student of Medicine** - 108

**Editor's Letter-Box** - 108

*NURSING SECTION.*

**Notes on News from the Nursing World—** Our Christmas Distribution — The Standard of Workhouse Nursing—Viceregal Visit to Sir Patrick Dun's Hospital—The Waltham Training School and Home Nursing—The late Sir George Gunning—The Club Question in Dublin—Nurses as Stewardesses—A New Matron at King's Lynn Hospital—Opening of a New Home at Grantham—The Nursing Staff at Camberwell Infirmary—Overcrowding in a Nurses' Home—The Modified Oath—District Nursing at Barnstaple—The Annual Sale at Peckham—Village Nurses in Cumberland—District Nursing at Cheadle—A School for Massage in Liverpool—Short Items - 75-78
**Lectures on Ophthalmic Nursing** - 79
**Workhouse Nursing** - 80
**National Union of Women Workers** - 81
**Appointments** - 82
**Presentations** - 83
**Everybody's Opinion** - 83
**Echoes from the Outside World** - 84
**A Book and its Story** - 85
**For Reading to the Sick** - 86
**Notes and Queries** - 86

